# Exploring the Antibiotic Potential of a Serine Protease from *Solanum trilobatum* Against *Staphylococcus aureus* Biofilms

**DOI:** 10.3390/idr17030050

**Published:** 2025-05-07

**Authors:** Manohar Radhakrishnan, Kanal Elamparithi Balu, Lakshminarayanan Karthik, Raghavendra Sashi Krishna Nagampalli, Eswar Kumar Nadendla, Gunasekaran Krishnasamy

**Affiliations:** 1 Department of Biochemistry and Molecular Biology, Indiana University School of Medicine, Indianapolis, IN 46202, USA; 2 Department of Biochemistry and Molecular Biology, University of Florida, Gainesville, FL 32611, USA; 3 Precision Therapeutics Laboratory, Quick IsCool, Aitele Research LLP, Bihar 843302, India; 4 Department of Immunology, St Jude Children’s Research Hospital, Memphis, TN 38105, USA; 5 CAS in Crystallography and Biophysics, University of Madras, Chennai 600025, India

**Keywords:** *Staphylococcus aureus*, biofilm, infectious disease, *Solanum trilobatum*, serine protease

## Abstract

Background: Multi-antibiotic resistance has become an alarming issue in treating bacterial infections in both community and medical environments. Globally, the scientific community has been exploring multi-antibiotic techniques to find new ways to address this challenge. To address this critical challenge and explore alternative antibiotic treatments, we investigated the potential of *Solanum trilobatum*, an edible and medicinally important herb plant in Ayurvedic medicine. Methods: Our research focused on a 60 kDa serine protease isolated and purified from the leaves of *S. trilobatum*, which showed evidence of possessing hydrolase activity. In this study, we examined the capability of the purified enzyme to eradicate preformed biofilms of *S. aureus* in combination with ampicillin. Additionally, we assessed the stability of the enzyme in the presence of metal ions and detergents. Results: Enzyme kinetics revealed a Vmax of 48.63 µM/min and a Km of 14.08 µM, indicating efficient enzymatic activity. Furthermore, the enzyme exhibited maximum activity at physiological pH, suggesting its potential effectiveness under physiological conditions. Conclusions: Our preliminary findings highlight the promising role of this enzyme as a potential agent to combat S. aureus biofilms, especially when used in conjunction with ampicillin, as an alternative antibiotic approach.

## 1. Introduction

A bacterial biofilm is a layer of bacterial colonization that forms beneath a protective wall made of the matrix. This protective wall consists of Extracellular Polymeric Substances (EPSs), which include proteins, lipids, polysaccharides, and extracellular DNA. These components play a vital role in developing various microbial infections [1]. Microbial cells within biofilms are known to survive extreme conditions and show resistance to factors such as UV radiation, metal-induced toxicity, exposure to acids, and changes in pH [2]. Additionally, EPS helps these pathogens evade the immune system by inhibiting neutrophil-mediated phagocytosis [3]. Furthermore, extracellular DNA (eDNA) and intercellular adhesins within EPS serve as barriers to antimicrobials, as eDNA can bind to human antimicrobial peptides (AMPs), reducing their effectiveness [4]. Moreover, many bacterial species have shown a greater tendency to accumulate on a variety of surfaces, eventually forming sedentary communities. These surfaces vary from plant and animal tissues to pipelines that carry household and industrial waste and prolonged usage of medical devices such as catheters, implants, and contact lenses [5]. Their proximity in these environments facilitates the efficient exchange of substrates, distributes the required metabolic products, and removes the toxic end products. This accumulation of mono- or poly-microbial aggregates results in the formation of biofilms comprising diverse communities of both bacteria and fungi [6]. Though they impart both positive and negative impacts on human health, biofilms are known to exhibit a profound effect in inducing several pathogenic forms of human diseases and plant infections. The most common examples are cystic fibrosis and dental plaque. While individuals affected by cystic fibrosis are susceptible to chronic *Pseudomonas aeruginosa* infections resulting in the formation of mucoid biofilm [7,8], patients affected by dental plaque may experience increased biofilm acidification, which, in turn, leads to the demineralization of the enamel, forming dental caries [9].

*Staphylococcus aureus* is widely recognized as a major human pathogen responsible for a variety of diseases, including skin infections, bacteremia, and infective endocarditis [10]. Among the different staphylococcal strains, *S. aureus* is considered the most virulent due to its diverse array of secreted and cell-surface-associated virulence factors, immune evasion mechanisms, and toxin production [11]. These strains exhibit significant genetic diversity, enabling them to form biofilms and acquire antibiotic resistance. Particularly concerning are the methicillin-resistant *S. aureus* (MRSA) strains, which often display multiple antibiotic resistance [12]. They are a leading cause of healthcare-associated infections, as they can attach to and accumulate on host tissues or medical devices, leading to complications such as surgical site infections, intravascular catheter (IVC) infections, and implant-related infections [11]. Research indicates that up to 80% of human bacterial infections are associated with biofilm-forming microorganisms like *S. aureus* [13]. Furthermore, *S. aureus* strains can also form biofilms in other sectors, such as the food and dairy industries. In these environments, they tend to adhere to milking machines and food-contact surfaces, showing high resistance to antibiotics and sanitization procedures [14]. These biofilms are particularly resistant to both innate and adaptive immune defense systems, as well as to antimicrobial agents, resulting in persistent infections and treatment failures. Owing to their widespread prevalence in diseases and prolonged resistance to antimicrobial treatments, exploring novel approaches to combat or prevent biofilm formation has become of paramount importance worldwide.

Proteases hydrolyze protein peptide bonds and can be classified as serine, cysteine, aspartic, metallo, glutamic, and threonine proteases based on their catalytic residue. Among all the known families, serine proteases are the most extensively characterized proteases [15]. They are involved in the metabolic processes of all organisms and regulate many physiological pathways. In general, proteases from leaves are few, and particularly, serine proteases from plant leaves are very rare [16,17]. Most of the characterized plant proteases are cysteine proteases, and they are readily reduced by air oxidation and metal ions [18]. The search for novel plant proteases with high stability has become a global research theme.

The plant *S. trilobatum* is well known for its traditional use in mucus clearance. Incidentally, this plant is known to demonstrate the existence of hydrolase. *S. trilobatum* has been in use for various ailments in different parts of Asian countries [19,20], and its active principle “sobatum” has been reported to possess anti-tumor activity [21,22] and anti-inflammatory activity [23], and the tannin of *S. trilobatum* leaves has been reported to have anti-bacterial activity [24,25]. The aqueous and methanol extracts of its leaves and stems were also reported to have anti-microbial activity [26]. The leaf extract of *S. trilobatum* was reported to exhibit anti-oxidant potency [27], as well as possess oviposition-deterrent and repellent activities against the mosquito *Anopheles stephensi* [28]. The presence of Nucleotide Binding Site (NBS)–Leucine-rich repeating (LRR) resistance gene analogs has also been reported in *S. trilobatum* plant species [29]. While plant extracts such as *Piper betle* L [30], *Eucalyptus globulus* Labill, and *Juglans regia* L [31] have been studied for their antibiofilm properties against *S. aureus* strains, a recent report demonstrated the anti-*Vibrio cholera* and *Staphylococcus aureus* action of a purified *S. trilobatum* antibacterial protein (*St*AP) from *S. trilobatum* leaves [25]; no published reports exist on the antibiofilm activity of *S. trilobatum* extracts. Furthermore, previous studies have primarily attributed the antibiofilm properties of these plant extracts to the presence of phytochemicals rather than proteins or enzymes. Therefore, this study presents a novel investigation into the potential of an *S. trilobatum*-derived serine protease in combating *S. aureus* biofilms. In this context, we describe the identification, purification, and biofilm-eradicating potency of a serine protease from the leaves of *S. trilobatum*, an edible plant known for its medicinal properties.

## 2. Materials and Methods

### 2.1. Extraction and Purification

As reported in an earlier study [25], the fresh leaves of *S. trilobatum* were collected from the Vandalur Forest area near Chennai, India. After removing the thorns, the leaves were thoroughly washed with distilled water and left to air dry. A total of 100 g of the dried leaves was weighed and then extracted using a 50 mL buffer containing 20 mM Tris (pH 7.8) and 150 mM NaCl. The resulting mixture was filtered through muslin cloth to remove large particulates.

The filtered extract was then centrifuged at 12,000 rpm for 40 min at 4 °C to separate the supernatant (crude extract) from the pellet. Enzymatic assays were performed on the supernatant using trypsin as the positive control and the extraction buffer as the negative control [15]. For further purification, the crude extract was subjected to ammonium sulfate precipitation. Pulverized ammonium sulfate was gradually added to the extract with constant stirring to achieve 30% saturation. After standing for 2 h at 4 °C, the mixture was centrifuged again at 12,000 rpm for 40 min at 4 °C. The resulting pellet was re-suspended in 3 mL of the extraction buffer. Subsequently, the saturation was adjusted to 30–60% and 60–80%, with the same centrifugation and re-suspension procedures applied for each fraction. The three obtained fractions were then extensively dialyzed against 20 mM Tris (pH 7.8) using 10 kDa cut-off dialysis membranes at 4 °C overnight, followed by an additional 4 h of dialysis exchanged with fresh buffer to remove excess salts.

### 2.2. Purification of Active Fraction via Anionic Exchange Chromatography

Ion exchange chromatography was performed using a strong anion-exchange column, Q-Sepharose HP-FF 1 mL (GE Life Sciences, Chicago, IL, USA), to purify the active 60–80% fraction. The column was pre-equilibrated with 10 column volumes (CVs) of buffer A [20 mM Tris, pH 7.8, containing 150 mM NaCl] and buffer B [20 mM Tris, pH 7.8, containing 1 M NaCl], at a flow rate of 1 mL/min, as mentioned by [25]. Before loading the active fraction, it was centrifuged briefly at 12,000 rpm at 4 °C to remove any particulate matter, and the clear supernatant was loaded onto the column. The unbound proteins were washed out with 8 CV of buffer A, while the bound proteins were eluted using a linear salt gradient (0 M NaCl to 1 M NaCl), collecting fractions in 20 CV of buffer B at a flow rate of 1 mL/min. The eluted and unbound fractions were analyzed for enzymatic activity to identify the fraction with the highest activity. Additionally, all fractions were subjected to Sodium Dodecyl Sulfate (SDS) polyacrylamide gel electrophoresis (PAGE) to assess their purity and homogeneity. This allowed for the identification of the fraction with the desired enzyme activity and ensured that the protein was adequately purified.

### 2.3. Biofilm Eradication Assay

The *S. aureus* strain from the Microbial Type Culture Collection and Gene Bank (MTCC) No. 96, obtained from the Institute of Microbial Technology (IMTECH), India, was grown overnight at 37 °C in autoclaved LB medium and then plated on an LB agar plate. A well-grown single colony was picked and inoculated into LB medium, where it was grown overnight at 37 °C with agitation at 180 rpm. These bacterial suspensions served as inocula for all biofilm experiments. The biofilm was formed by adding 900 µL of LB medium to 100 µL of the *S. aureus* inoculum, mixed in two different 24-well polystyrene plates from top to bottom in their respective wells. The wells were sealed with transparent tape and incubated at 37 °C for 2 days under static conditions. After 2 days, the contents of the wells were carefully discarded and washed once with a 0.1 M NaCl solution, followed by three washes with water. The wells of the first plate alone were stained with a crystal violet solution for 15 min and then washed with water. The formed biofilms were observed using an optical microscope. In the second plate (unstained), the preformed biofilm was treated with 160 µM of purified protease as well as a combination of the protease fraction and ampicillin and kept overnight at 37 °C in a static environment. After 12 h, the contents of the well were carefully discarded and washed three times with water. The wells were stained with a crystal violet solution, and then, they were washed with water. The changes in the biofilms were analyzed with the help of an optical microscope, and the experiment was adopted with slight modification from Jayarajan, S. et al. [32].

### 2.4. Proteolytic Activity Measurement

Protease activity was measured using a modified UV spectroscopy method based on Benyon [33]. A 160 µM aliquot of the protease fraction was mixed with an equal volume of 1% casein (*w*/*v*) and incubated at 37 °C for 30 min. The reaction was terminated by adding 200 µL of 10% ice-cold trichloroacetic acid (TCA), and the mixture was incubated for an additional 30 min at 37 °C. After incubation, the sample was centrifuged at 10,000 rpm for 30 min at 4 °C to separate the precipitate. The clear supernatant, containing the lytic fragments of casein, was collected and analyzed for absorbance at 280 nm using a UV spectrophotometer [34]. Proteolytic activity was assessed for both the aqueous crude extract and ammonium sulfate fractions, with positive (trypsin) and negative (lysis buffer used in protein preparation) controls included for comparison.

### 2.5. Effect of Standard Inhibitors on Protease Activity

The aqueous extract, purified protease, was assessed for its proteolytic activity using the casein agar radial diffusion method [15]. To determine the protease’s characteristic nature, specific inhibitors were used: Ethylenediaminetetraacetic acid (EDTA) for metalloproteases, Iodoacetic acid (IAA) for cysteine proteases, and phenylmethylsulphonyl fluoride (PMSF) for serine proteases. EDTA and IAA were dissolved in water, while PMSF was dissolved in 10% ethanol. The purified protease was pre-incubated with 5 mM EDTA, 2 mM IAA, and 2 mM PMSF in separate vials; the reaction mixture was added into the respective wells of the casein agar plate kept at 37 °C overnight; and the proteolytic activity was then measured from the digestion of the casein zone.

### 2.6. Effect of Detergents, Organic Solvents, and Metal Ions on Proteolytic Activity

The stability of the protease was evaluated in the presence of various organic solvents such as toluene, benzene, acetone, isopropanol, surfactants, and an oxidizing agent. The reaction mixture, consisting of purified protease and a 1% casein solution (pH 7.8), was pre-incubated with 0.1 mM of various metal ions, including Ca^2+^, Zn^2+^, Cu^2+^, Mg^2+^, Mn^2+^, Fe^2+^, and Ba^2+^, at 37 °C for 30 min. The reaction was then stopped by adding 10% trichloroacetic acid (TCA) and incubated at room temperature for 30 min with slight modification [34,35]. Afterward, the mixture was centrifuged at 10,000 rpm for 20 min to separate the precipitate. The supernatant was mixed with 0.5 M sodium carbonate and Folin–Ciocalteu reagent and incubated at room temperature for 30 min. The amount of tyrosine released from casein was quantified by measuring the absorbance at 660 nm. A control assay was performed without metal ions, and the resulting enzyme activity was considered 100%. The effect of each metal ion on protease activity was determined by comparing the enzyme activity in the presence of the metal ions to the control. The same methodology was followed to determine the effect of SDS, 0.2% Triton X-100, and Tween 80, along with 0.2% H_2_O_2_ as the oxidizing agent [36].

### 2.7. Effect of pH and Temperature on Proteolytic Activity

To assess the effect of pH on the purified protease, the enzyme was exchanged into buffers with varying pH levels (4 to 9): 20 mM sodium acetate buffer (pH 4.0 and 5.0), 20 mM sodium phosphate buffer (pH 6.0 and 7.0), and 20 mM Tris buffer (pH 8.0 and 9.0). The exchange was carried out for 12 h at 4 °C by dialysis, after which the enzymatic activity was measured using a previously described method with some modifications [15]. The effect of temperature on enzymatic activity was determined by incubating the purified protease at different temperatures (20 °C, 40 °C, 60 °C, and 80 °C) for 30 min in 20 mM Tris buffer (pH 7.8). After incubation, proteolytic activity was assessed by measuring the zone of casein digestion as mentioned earlier.

### 2.8. Zymography

Zymography was performed at 4 °C using 1% casein and gelatine as a substrate, which was copolymerized with polyacrylamide gel. After electrophoresis under non-reducing conditions at 50 mV, the gel was washed twice with 50 mL of renaturation buffer (2.5% Triton X-100) for 2 h to remove SDS. The gel was then incubated overnight at 37 °C in a buffer containing 20 mM Tris (pH 7.8), 50 mM NaCl, and 10 mM CaCl₂. After incubation, the gel was stained with Coomassie Brilliant Blue G-250. The proteolytic activity of the enzyme was visualized as a clear, colorless zone against the substrate background, indicating gelatin and casein digestion [37].

### 2.9. Determination of Vmax and km

The spectroscopic method was employed to determine the kinetic parameters, Vmax and Km, of *S. trilobatum* serine protease using casein as the substrate, as described with a slight modification [38]. A fixed concentration of 5 mM serine protease, along with a fixed reaction time, was used to measure the rate of proteolysis as the substrate concentration increased. To assess residual protease activity, varying concentrations of casein (ranging from 7 to 80 µM) were added to purified serine protease and incubated for 30 min at 37 °C in a 50 mM Tris buffer at pH 7.8. The reaction was stopped by adding 10% ice-cold TCA solution after the 30 min mark. After incubation at 4 °C for an additional 30 min, the mixture was centrifuged at 12,000 rpm for 30 min, and the supernatant was used for the spectroscopic analysis. The progression of the proteolysis reaction was observed by measuring UV absorbance at 280 nm. The Km and Vmax value of serine protease was determined by the substrate–velocity curve. Assays were carried out in triplicate, and the kinetic parameters were determined using GraphPad Prism 6.0 (Graphpad.com, San Diego, CA, USA).

## 3. Results and Discussion

### 3.1. Isolation and Purification of Protease from Solanum Trilobatum

**Figure 1:**
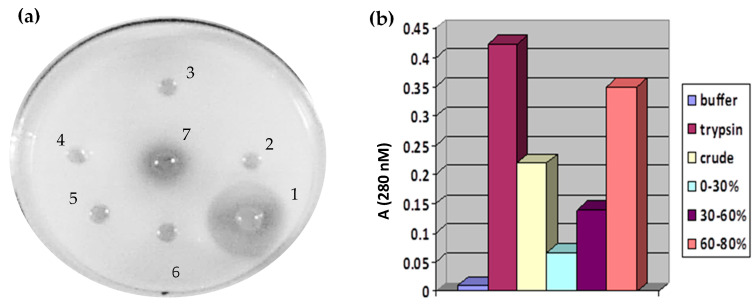
(**a**) Caseinolytic activity of the aqueous crude extract (1—positive control; 2–6—negative controls), well No. (1) 2 mM trypsin, (2) 20 mM Tris (7.8) + 150 mM NaCl (extraction buffer), (3) 150 mM NaCl, (4) 20 mM Tris (7.8), (5) water, (6) 1 M NaCl, and (7) crude extract *S. trilobatum* 50 μL. (**b**) Serine protease activity of the dialyzed fractions was obtained from a UV spectroscopic assay, where casein was used as the substrate.

The *S. trilobatum* leaves were extracted in a 20 mM Tris and 150 mM NaCl buffer, following which the protease activity of the crude extract was tested by the radial diffusion method (Figure 1a). By using casein as the substrate, the dialyzed ammonium sulfate fractions (0–30%, 30–60%, and 60–80%) along with the aqueous crude extract were evaluated by the UV spectroscopy method. The proteolytic activity was measured from the cleaved and liberated casein fragments, which were subsequently quantified by UV 280 nm absorbance. Among the various fractions, the 60–80% fraction exhibited maximum protease activity similar to that of bovine trypsin (Figure 1b). Substantial protease activity was also observed in the crude extract.

**Figure 2:**
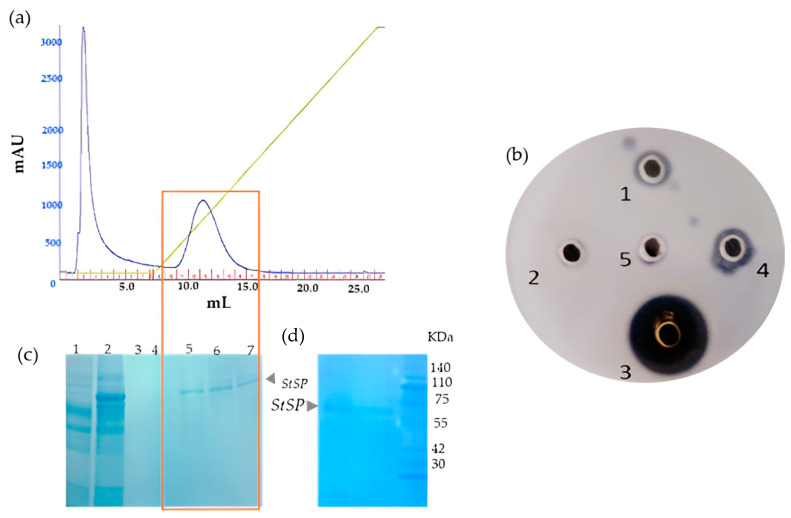
(**a**) Purification of *St*SP: Ion exchange chromatography (IEC) of the 60–80% fraction. The green line represents the rise of salt concentration by a linear gradient from 0 M to 1 M NaCl. (X-axis: 1 mL/min, Y-axis: UV 280 nm). (**b**) Caseinolytic activity of IEC fractions**:** well No. (1) IEC elution tube number 12, (2) IEC flow-through fraction, (3) 2 mM trypsin (positive control), (4) IEC elution 13, (5) IEC elution buffer 20 mM Tris pH 7.8 + 1 M NaCl (negative control). (**c**) IEC fractions on 10% SDS PAGE: lane No. (1) IEC flow-through, (2) 60–80% ammonium sulfate fraction, elution 10, (4) IEC elution 11, (5) IEC elution 12, (6) IEC elution 13, (7) IEC elution 14. (**d**) Purified *St*SP with standard protein ladder.

Based on this observation, the active fraction (60–80%) was loaded onto a Q Sepharose Fast Flow anionic exchange column. While the unbound proteins were collected as an unabsorbed fraction with the same buffer A [20 mM Tris, pH 7.8, containing 150 mM NaCl], the bound proteins were eluted using an increasing salt concentration, starting from 0 M NaCl to 1 M NaCl. The elution profiles of both the flow-through and active fractions are depicted in Figure 2a. Following the chromatographic run, both the unbound and elution peak fractions were analyzed for their proteolytic activity on a casein agar plate. The activity study revealed that only the eluted peak (bound) fractions exhibited reliable protease activity when compared to the unbound fraction (Figure 2b). Using 10% SDS-PAGE, the homogeneity of those bound fractions displaying the caseinolytic activity was determined, confirming the protease’s purity (Figure 2c). Interestingly, the molecular weight (~60 kDa) of this purified *S. trilobatum* serine protease (*St*SP) was found to be in accordance with serine proteases found in the fruits of *Cucumis trigonaus* [39], the leaves of wheat seeds [40], and the seeds of *Solanum dubium* [41].

As a result, the fractions exhibiting proteolytic activity were pooled, concentrated, and used for further kinetic as well as other functional characterization studies. The proteolytic activity of this *St*SP was then evaluated again by UV spectroscopy using casein as the substrate (Figure 2d).

### 3.2. Inhibition of Serine Protease by Serine Protease Inhibitor (PMSF)

**Figure 3:**
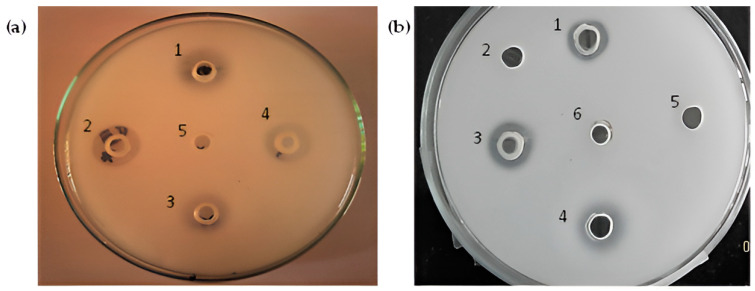
(**a**) Effect of metalloprotease inhibitor (EDTA) and cysteine protease inhibitor (IAA): well No. (1) 100 µg purified protease incubated with 5 mM EDTA, (2) 100 µg trypsin (positive control), (3) 100 µg purified protease incubated with 2 mM IAA, (4) 100 µg purified protease, (5) IEC buffer (negative control). (**b**) Effect of serine protease inhibitor (PMSF): well No. (1) 100 µg purified protease, (2) 100 µg purified protease pre-incubated with 2 mM PMSF, (3) 100 µg trypsin (positive control), (4) 100 µg purified protease, (5) 10% ethanol (negative control), (6) 20 mM Tris pH 7.8 + 150 mM NaCl buffer (negative control).

As outlined in the methodology section, the enzyme activity of the *St*SP was tested in the presence of the cysteine protease inhibitor Iodoacetamide (IAA), the metalloprotease inhibitor (Ethylenediaminetetraacetic acid—EDTA), and the serine protease inhibitor (Phenylmethylsulfonyl fluoride—PMSF). The results indicated that protease activity remained unaffected in the presence of either EDTA or IAA, as evidenced by distinct zones of digestion observed in the wells containing these inhibitors (Figure 3a). Conversely, the incorporation of the serine protease inhibitor PMSF into the purified protease completely inhibited its enzymatic activity (Figure 3b), confirming that the *St*SP is a member of the serine protease family.

### 3.3. Staphylococcus aureus-Preformed Biofilm Eradicated by StSP

**Figure 4:**
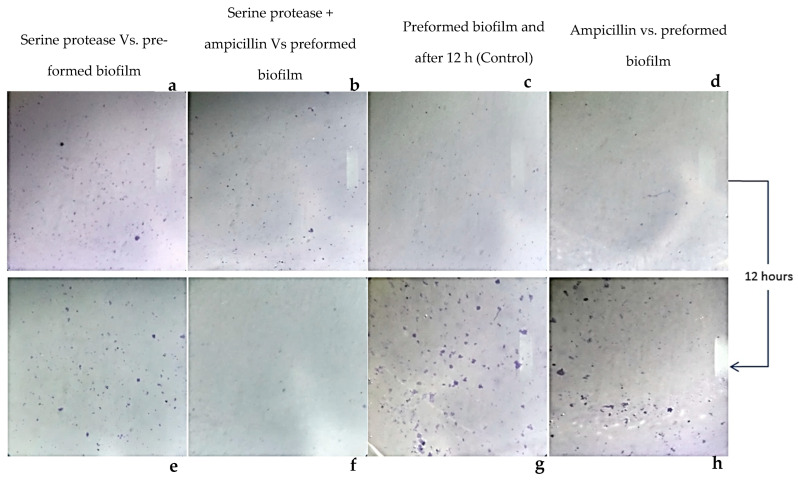
The biofilm eradication potency of *St*SP. The control panels (**c**) and (**g**) show the *S. aureus-*preformed biofilm on the surface of the microtiter plate at the initial time point before and after 12 h of incubation. The samples’ (serine protease, serine protease + ampicillin, ampicillin) treated panels are shown in (**a**,**b**,**d**), respectively. The eradication potency of *St*SP samples after 12 h of treatment was observed on the panels (**e**–**h**).

Biofilm eradication experiments were employed to understand the effect of these enzymes on preformed biofilm surfaces. The experiments reveal the destruction of preformed biofilm by treatment with these enzymes. In Figure 4, the upper panels (a, b, c, and d) show the preformed biofilm on the polystyrene surface of the microtiter plate. The lower panels (e, f, g, and h) correspond to the respective sample treatments mentioned in the figure. In the lower lane of the figure, the *St*SP-treated biofilm shows reduced growth compared to the control. When the preformed biofilm was incubated with media and grown statically, profound propagation was witnessed (referred to as “untreated”). The robustness of the biofilm could be witnessed by the fact that ampicillin had no effect on the biofilm and the propagation was as good as the untreated surface (Figure 4h). Though they differ in their specificities, the degree of disturbance exhibited by both the enzyme and the combination of the enzyme and the antibiotic (*St*SP and ampicillin) is comparable. From a visual comparison of the *St*SP and the combination of the enzyme and the antibiotic, the combined usage was found to have better biofilm eradication potency than the simple enzyme (*St*SP) treatment (Figure 4f). The presence of the biofilm even after treatment with enzymes may be due to the spread of biofilm fragments, which are cleaved off by enzymes. The reduced biofilm growth suggests the influence of antibiotics on the fragment or the planktonic bacteria excreted from the biofilm after the digestion of EPS.

### 3.4. Effect of Metal Ions

**Figure 5:**
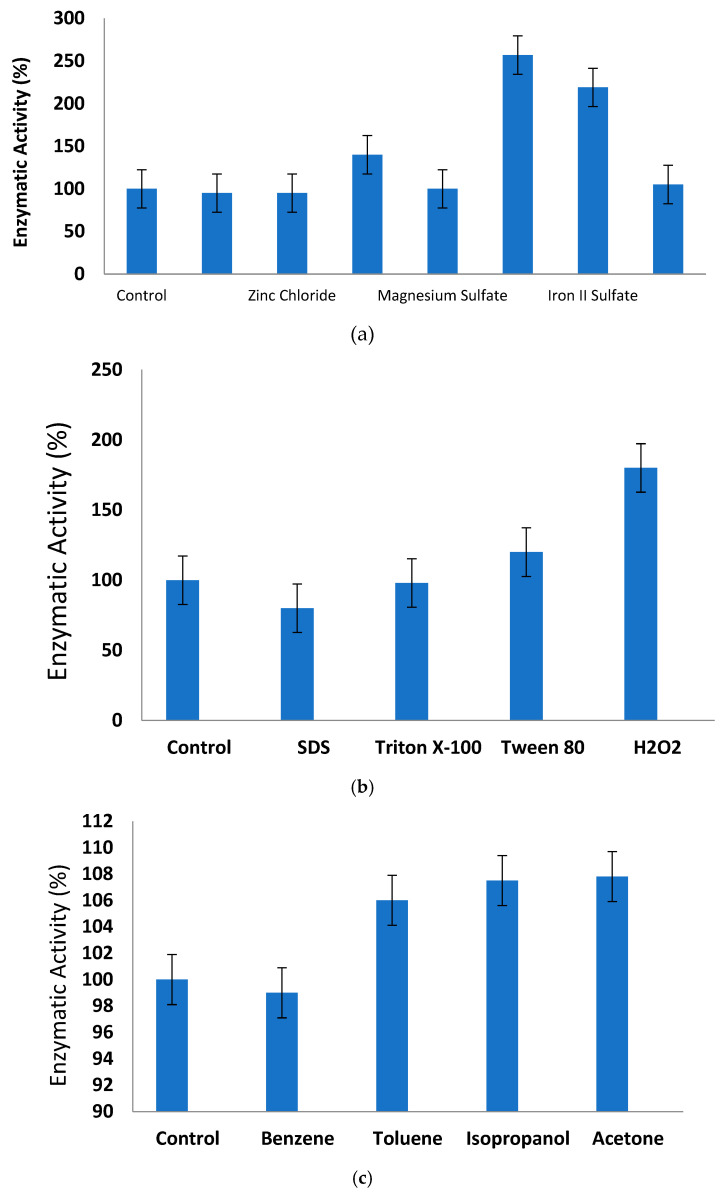
(**a**) Effect of metal ions on *St*SP. (**b**) Effect of various surfactants on *st*SP. (**c**) Effect of various organic solvents on *st*SP.

The enzymatic activity of the serine protease isolated from *S. trilobatum* leaves exhibited a significant enhancement with the addition of 0.1 mM Mn^2+^ and Fe^2+^ metal ions, while experiencing a moderate activation with Cu^2+^ and Ba^2+^ (Figure 5a). Notably, the residual activity showed a remarkable increase of more than 1-fold, reaching up to 1.5-fold and 1.1-fold when incubated with Mn^2+^ and Fe^2+^ metal ions, respectively. However, the enzymatic activity of the *St*SP remained unaffected by the presence of Zn^2+^ and Ca^2+^ ions among the tested metal ions (Figure 5a). Interestingly, a similar activation of residual activity by a 60 kDa serine protease from pigeon pea leaves was observed with Mn^2+^, Fe^2+^, and Cu^2+^. In summary, none of the tested metal ions inhibited or altered the proteolytic activity of the *St*SP, aligning with previously published reports that emphasize the role of metal ions in enhancing the activity of plant proteases [42].

### 3.5. Effect of Surfactants

The presence of 5 mM SDS and 0.2% Triton X-100 minimally impacted the activity of the *St*SP, while the addition of 0.2% Tween resulted in a notable 0.2-fold enrichment in activity (Figure 5b). This finding aligns with previous studies on a 30 kDa serine protease from *Scorzonera hispanica* [43], where the proteolytic activity of the *St*SP exhibited noteworthy resistance to these detergents. Particularly interesting is the fact that a 0.8-fold excess of the activity was retained when incubated with 0.2% of the oxidizing agent H_2_O_2_. The resistance of *S. trilobatum* protease to these detergents and oxidizing agents opens new possibilities for its application in detergent formulations.

### 3.6. Effect of Organic Solvents

Enzymes often experience instability in the presence of various organic solvents, leading to denaturation. The stability of the *St*SP was assessed in the presence of different organic solvents, including toluene, benzene, acetone, and isopropanol (Figure 5c). However, when treated with acetone, the activity was retained at more than 1-fold, and it approached the same fold when exposed to isopropanol as toluene. Consequently, the enzyme exhibited greater stability in acetone. The enzyme’s stability in these solvents is comparable to that reported for serine proteases from *Scorzonera hispanica* [43].

### 3.7. Determination of Kinetic Parameters

**Figure 6:**
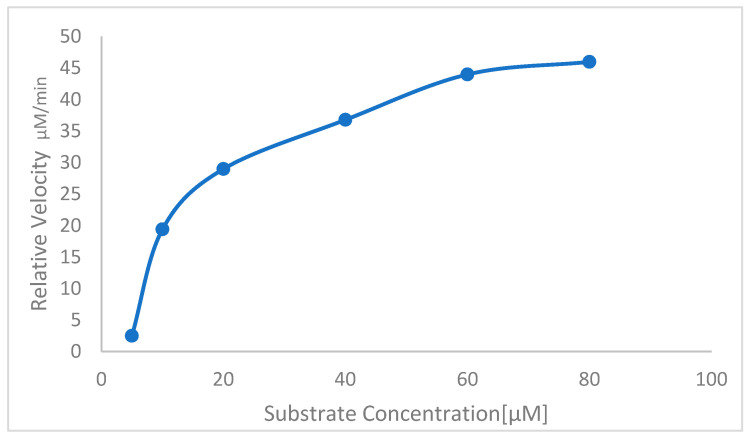
Determination of kinetic parameters, Vmax and Km, of the purified *st*SP.

UV-based enzyme–substrate kinetic parameters Vmax and Km for the purified *St*SP were determined by using casein as the substrate. The substrate–velocity curve yielded Vmax and Km values of 48.63 µM/min and 14.63 µM, respectively (Figure 6).

The determined Km value reflects the *St*SP’s affinity towards the serine protease substrate, while Vmax signifies the enzyme’s maximum catalytic ability, aiming for optimal efficiency. The study involved varying substrate concentrations (7–80 µM), revealing that, at higher concentrations of the substrate, the proteolytic activity reached the enzyme’s saturation point. This was evident in the linear regression plot and the Km of this enzyme, at 14.63 µM. The *St*SP enzyme has a Vmax of approximately 48.63 µM and a Km value of 14.08 µM/min. This suggests that its catalytic function is more efficient than that of a 69.9 kDa serine protease found in the seeds of *Cyamopsis tetragonoloba*, which has a Vmax of about 102 µM and a Km of approximately 56.56 µM/min [44]. Additionally, there is a stable 57.9 kDa serine protease derived from the latex of *Wrightia tinctoria* [45], which has a Km value of around 50 µM. Furthermore, a subtilisin-like serine protease, weighing about 59 kDa and reported from wheat leaves, exhibits a Vmax of approximately 2.27 mM and a Km of 1.18 mM [40].

### 3.8. Sustainability of Various pH Ranges and Temperatures

**Figure 7:**
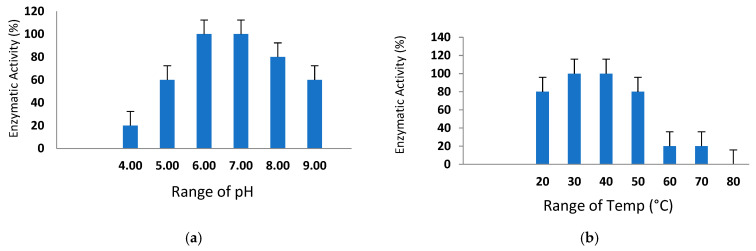
(**a**,**b**) Effect of pH and temperature.

The pH and temperature stability of the purified *St*SP were assessed across various ranges (pH 4.0–9.0) and temperatures (20 °C to 80 °C) using casein as the substrate, employing the radial diffusion method. As depicted in Figure 7a, the protease activity exhibited a gradual increase from an acidic pH of 4.0 to a maximum at a neutral pH. The optimal pH for enzymatic activity was determined to be 7.0, with a functional range identified between pH 6 and 8. The caseinolytic activity of the protease remained robust within a narrow temperature range of 40 °C to 50 °C, retaining full functionality. Up to 50 °C, the activity was nearly constant, but a sudden decline occurred at 60 °C and 70 °C, as illustrated in Figure 7b, and at 80 °C, the caseinolytic activity was completely lost. This loss may be attributed to a slight conformational change in the active or binding site, leading to the loss of proteolytic activity.

The moderate thermal stability and optimal activity at pH 7.0 observed in this study are consistent with other serine proteases reported for *Kesinai* leaves [46].

**Figure 8:**
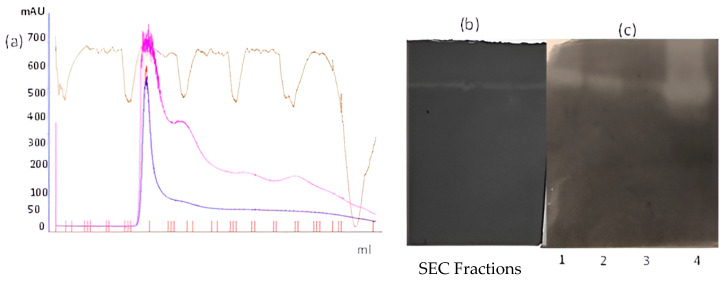
(**a**) Size-exclusion chromatography of serine protease on G-100 Sephadex resin. (**b**) Caseinolytic activity of size-exclusion fractions on zymogram. (**c**) Zymography: lane (1) purified serine protease, (2) 60–80% ammonium sulfate fraction, (3) aqueous extract of *S. trilobatum* leaves, (4) purified and concentrated *St*SP.

The proteolytic activity of the purified protein was confirmed through zymography on the background using both gelatin (Figure 8b) and casein (Figure 8c) as substrates. Clear zones of digestion with those substrates were observed, as witnessed under the non-denaturing conditions as a single band of digestion. In Figure 8b, the size-exclusion fractions from Figure 8a correlate with the SEC peak, indicating the enzyme exists as a monomer in native, non-denaturing, and denaturing systems.

## 4. Conclusions

To the best of our knowledge, this is the first purification and characterization report on a 60 kDa serine protease from the leaves of *S. trilobatum*. The consumable nature and medicinal value of these leaves, coupled with their therapeutic properties against respiratory ailments, suggest potential biological relevance in human health. While these leaves are already consumed as fresh juice and as a food additive, their newfound potential presents an innovative direction for the industry. The purified enzyme showcases proteolytic action, with an optimum pH of 7.0 and moderate thermal stability up to 50 °C. The purified *St*SP displayed the capability to eradicate biofilms produced by *S. aureus*, thus supporting the traditional use of the leaves for alleviating throat infections and gastrointestinal issues, as well as reducing mucus congestion.

## Data Availability

The original data presented in the study are openly available at https://www.preprints.org/manuscript/202504.0509/v1 (accessed on 22 April 2025).
